# Facet-joint injections for people with persistent non-specific low back pain (FIS): study protocol for a randomised controlled feasibility trial

**DOI:** 10.1186/s13063-015-1117-z

**Published:** 2015-12-24

**Authors:** Harbinder Sandhu, David R. Ellard, Felix Achana, James H. L. Antrobus, Shyam Balasubramanian, Sally Brown, Melinda Cairns, Frances Griffiths, Kirstie Haywood, Charles Hutchinson, Ranjit Lall, Stavros Petrou, Nigel Stallard, Colin Tysall, David A. Walsh, Martin Underwood

**Affiliations:** Warwick Clinical Trials Unit, Division of Health Sciences, Warwick Medical School, The University of Warwick, Coventry, CV4 7AL UK; Anaesthesia & Pain Medicine, South Warwickshire NHS Foundation Trust, Warwick Hospital, Lakin Road, Warwick, CV34 5BW UK; Anaesthesia & Pain Services, University Hospital Coventry and Warwickshire, Clifford Bridge Road, Coventry, CV2 2DX UK; UNTRAP, The University of Warwick, Coventry, CV4 7AL UK; Department of Allied Health Professions and Midwifery, School of Health and Social Work, University of Hertfordshire, Hatfield, Hertfordshire, AL10 9AB UK; Social Science and Systems in Health, Division of Health Sciences, Warwick Medical School, The University of Warwick, Coventry, CV4 7AL UK; RCN Research Institute, Division of Health Sciences, Warwick Medical School, The University of Warwick, Coventry, CV4 7AL UK; Population Evidence and Technologies Room, Warwick Medical School, The University of Warwick, University Hospitals of Coventry and Warwickshire, Clifford Bridge Road, Coventry, CV2 2DX UK; Statistics and Epidemiology, Division of Health Sciences, The University of Warwick, Coventry, CV4 7AL UK; Arthritis Research UK Pain Centre, Academic Rheumatology, University of Nottingham, NG51PB, Nottingham, UK

**Keywords:** Intra-articular facet joint injections, Low back pain, Combined physical and psychological programme, Corticosteroids

## Abstract

**Background:**

The role of injections of therapeutic substances into the back as treatment for low back pain is unclear. Facet joint injections are widely used despite the absence of evidence of sustained benefit. We hypothesise that facet joint injections might facilitate engagement with physiotherapist-led, best usual care (a combined physical and psychological programme) and is a clinically and cost-effective treatment for people with suspected low back pain of facet joint origin.

**Methods/Design:**

We present here the protocol for a randomised controlled feasibility trial for a main trial to test the above hypotheses.

Patients referred to secondary care with persistent non-specific low back pain will be screened and invited to take part in the study. Those who meet the eligibility criteria will be invited for a physiotherapy assessment to confirm trial eligibility and for baseline data collection. All participants (*n* = 150) will be offered the best usual care package with physical and psychological components. Those randomised into the intervention arm (*n* = 75) will, in addition, receive intra-articular facet joint injections with local anaesthetic and steroids. Primary outcome data will be collected using daily and then weekly text messaging service for a pain score on a 0–10 scale. Questionnaire follow-up will be at 3, 6, and 12 months.

Evaluation of trial processes and health economic analyses, including a value of information analysis, will be undertaken. The process evaluation will be mixed methods and will include the views of all stakeholders.

**Discussion:**

Whilst this trial is a feasibility study it is currently one of the largest trials in this area. The outcomes will provide some evidence on the use of facet joint injections for patients with clinically diagnosed facet joint pain.

**Trial registration:**

EudraCT identifier 2014-000682-50, (registered on 12 February 14). ISRCTN registry number: ISRCTN93184143 DOI 10.1186/ISRCTN93184143 (registered on 27 February 2014).

**Electronic supplementary material:**

The online version of this article (doi:10.1186/s13063-015-1117-z) contains supplementary material, which is available to authorized users.

## Background

Low back pain is a common and costly problem. People with chronic disability use the majority of the National Health Service (NHS) and other resources that are devoted to back pain [1]. Guidelines from the National Institute for Health and Care Excellence (NICE) for the management of non-specific low back pain were published in 2009. These proposed a care pathway in which all those people with non-specific low back pain that has not resolved after 6 weeks should be offered a choice of three different therapist-delivered interventions; exercise, or manual therapy, or acupuncture [2, 3]. Those with continuing problems after one or more of these treatment options would then be able to access an intensive programme of combined physical and psychological intervention lasting up to 100 hours, a duration that has rarely been delivered in practice. The guidelines made ‘do not use’ recommendations for a range of treatment approaches that have not been proven to be effective through randomised controlled trials, including the injection of therapeutic substances into the back. Some of these procedures, including intra-articular facet joint injections, whilst not universally available, remain in common use [4]. There has been renewed interest in evaluating invasive procedures for low back pain including intra-articular facet joint injections [5–7]. A definite diagnosis of facet joint pain can only be made following a diagnostic procedure. In clinical practice it would be unusual to do a diagnostic procedure before considering a therapeutic intra-articular facet joint injection; the diagnosis of probable facet joint pain is usually made on clinical grounds alone.

Continuing pain, or increasing pain during physiotherapy or other exercise, is cited as a barrier to engagement with physiotherapy interventions [8]. Even short-term pain relief following facet joint injections might facilitate rehabilitation and improve patient outcomes. We, therefore, wish to conduct a randomised controlled trial of adding intra-articular facet joint injections to a combined physical and psychological intervention, deliverable in the NHS, for people with living with probable facet joint pain. If the trial has positive results then use of intra-articular facet joint injections will be justified. If the trial is negative its conclusions need to be sufficiently robust that all parties are satisfied that the evidence does not support their use. Here we present the protocol for a feasibility trial for such a trial [9]. This protocol has been developed and informed by recent best evidence and a consensus conference of experts [4].

## Methods

### Objectives of the trial

#### Primary objective

The primary objective of this trial is to explore the feasibility of running a randomised controlled trial to test the hypothesis that for people with suspected facet joint pain contributing to persistent low back pain, the addition of intra-articular facet joint injections to best usual non-invasive care available from the NHS is clinically and cost effective.

### Secondary objectives

- To test and evaluate agreed criteria for identifying people with suspected facet joint pain.
- To test and evaluate an agreed protocol for the injection of facet joints in a consistent manner.
- To test and evaluate a standardised control treatment deliverable in the NHS and congruent with NICE guidance (best usual care).
- To test systems for collecting short- and long-term pain outcomes, including measures required for economic evaluation.
- To demonstrate that recruitment to the main trial is feasible.
- To collect the recruitment and outcome data required to inform sample size and number of sites needed for the main trial.
- To obtain an initial indication of pain relief to inform the need for a full trial.
- To do a process evaluation that includes patient experience within the trial.

### Trial design

This is a multicentre (up to six sites) randomised controlled trial with two arms. All participants will be offered a bespoke combined physical and psychological rehabilitation package. Those randomised into the intervention arm will, in addition, receive intra-articular facet joint injections with local anaesthetic and steroids.

### Participants and setting

Patients referred from primary care to a secondary care NHS Trust (Trust) who have chronic low back pain will be invited to screening for potential inclusion. We will also test the feasibility of setting up a general practitioner (GP) and community physiotherapy referral system into a secondary care physiotherapist-led research clinic. This will aim to recruit those back pain patients seen in primary and community care with substantial problems after simple interventions.

#### Inclusion criteria

1. Able and willing to comply with the trial procedures and signed and dated informed consent is obtained;
2. Aged >18;
3. Has at least moderately troublesome low back pain present for at least 6 months [10];
4. Has low back pain as their predominant musculoskeletal pain;
5. Has undergone registered health professional-delivered treatment for low back pain in the preceding 2 years prior to inclusion;
6. Meets clinical criteria for suspected facet joint pain AND no radicular symptoms (defined as pain radiating below the knee) AND no sacro-iliac joint pain elicited using a pain provocation test AND increased pain unilaterally, bilaterally on lumbar para-spinal palpation, AND increased low back pain on one or more of the following; extension (more than flexion), rotation, extension/side flexion, extension/rotation;
7. Is able to use text messaging, or an alternative means of daily data collection (paper-based diary);
8. Is fluent in written and spoken English.

#### Exclusion criteria

1. Unable to attend for randomised treatment, or other circumstances that would significantly decrease the chance of obtaining reliable data, achieving trial objectives or completing the trial and follow-up assessments or is considered unsuitable to participate in the trial by an investigator;
2. Is unable/unwilling to undergo injections;
3. Has used oral corticosteroids or had a corticosteroid injection in the preceding 3 months;
4. Has an underlying serious psychiatric or psychological disorder that precludes participation in either intervention;
5. Has previously undergone spinal injections;
6. Has previously undergone spinal surgery;
7. Has a contraindication to facet joint injections for example a serious co-morbidity (e.g. severe chronic obstructive pulmonary disease (COPD), poorly controlled diabetes), malignancy, infection, inflammatory disorder, or fracture, or is taking anti-coagulant medications;
8. Has a known allergy to the constituents of the planned injections;
9. Pregnancy, or suspected pregnancy;
10. Was previously randomised in this trial;
11. Is currently participating in another clinical trial (with an unregistered medicinal product), or less than 90 days have passed since completing participation in such a trial.

### Consent

**Fig. 1:**
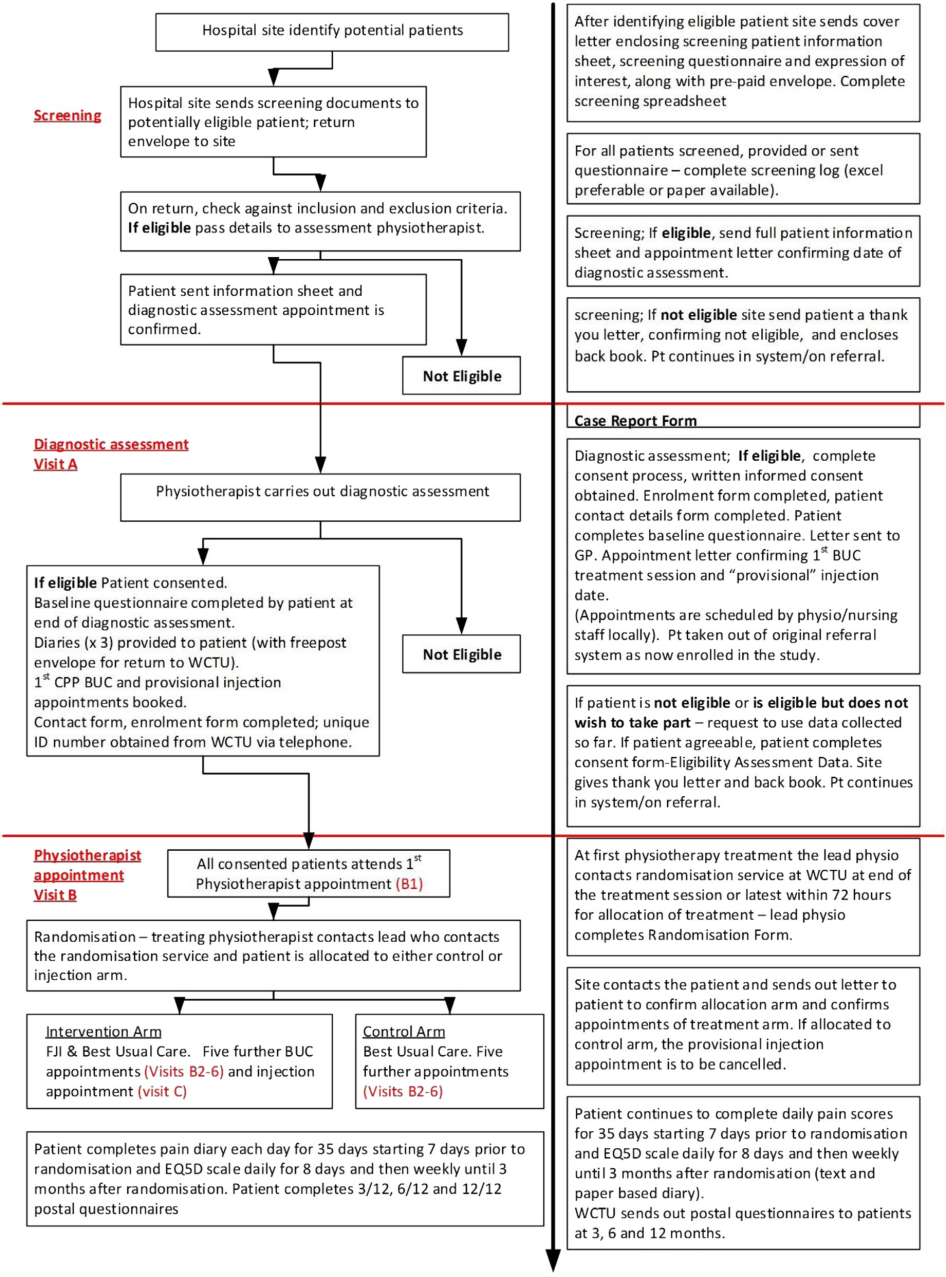
Patient flow chart and associated forms

All potential participants, identified from referrals to secondary care or secondary care clinics, will receive information about the study and a brief screening questionnaire. If they are potentially eligible they will be provided with additional information. An assessment consultation with a research physiotherapist will determine if a participant is actually eligible. If they are eligible and they would like to join the study, written informed consent will be obtained by trained research health professionals at each of the sites. The patient flow is illustrated in Fig. 1. For participants who are to receive an injection, the injecting clinician will confirm suitability and obtain informed written consent for the procedure adhering to normal practice for their institution.

### Power and sample size

There are several drivers for the sample size for this feasibility trial; including, estimating the proportion of patients who gain immediate pain relief, and obtaining sufficient outcome data to inform a decision to proceed. We will recruit 150 participants to the randomised feasibility trial over 6 months, randomised into two equal groups. Data from the resulting 75 patients in the active injection group will allow us to estimate the proportion with ‘true’ facet joint pain, based on achieving immediate pain relief (a reduction of 50 % or more), with a standard error of 5.5 % if the true proportion is 62 %. The proportion of 62 % is based on the figure found in the best previous study; we would hope we can achieve a higher treatment response rate than this with a commensurate increase in precision.

We have also considered how the effect size found in a between-group comparison within this feasibility trial will influence a decision to run a main trial. Essentially, we should only proceed if the limit for the 95 % confidence interval includes a value that would be indicative of a clinically important difference in favour of the injection group; if it does not then we should not proceed. At the design stage we calculated that if the desired standardised mean difference (between-group difference/pooled baseline standard deviation) indicative of a minimally clinically important difference is in range 0.3–0.4, then after allowing for 20 % loss to follow-up, if we recruit 150 participants then the probability of proceeding to a full trial if true effect is zero is around 50 %. Further details of the sample size calculation are provided as a Additional file 1.

### Randomisation

Participants will be randomised sequentially, using minimisation to balance randomisation with NHS Trust (i.e. the NHS area in the UK where the treating hospital is based), participant age group and troublesomeness of low back pain. Participants will be randomised to receive facet joint injections combined with ‘best usual care’ or ‘best usual care’ only.

Randomisation will be performed centrally by Warwick Clinical Trials Unit (WCTU) using a remote telephone randomisation system to ensure concealment and avoidance of bias.

### Outcome measures

Table 1 below summarises the outcome measures and their time of completion by participants. The main questionnaire packages are completed at baseline (at trial entry assessment) and follow-up (3, 6 and 12 months post randomisation). A pain severity (today) score will be recorded daily for 35 days from 7 days before the first treatment session and following this weekly until 3-month follow-up. For this we are testing the use of text messaging to allow participants to respond by text. However, paper diaries will be provided as a back-up as this is our primary outcome. Health utilities (measured using the EuroQol EQ-5D-5 L) will be recorded weekly from 1 week prior to the first treatment session until the night before the injection appointment when we will ask participants to complete the EQ-5D-5 L measure for 8 days then weekly until the 3-month follow-up. In addition, intervention participants will record a pain severity score 45–60 minutes before and after injection. Clinical data will be collected and recorded by physiotherapists and clinicians, covering patients’ assessments, injections and involvement in the best usual care. It may be necessary to increase the number of data points for the EQ-5D-5 L and the pain severity score; first, if an injection is cancelled/rescheduled and second, towards the end to ensure test-retest reliability. We will undertake postal follow-up with two postal reminders, and one telephone call for primary outcomes at 3, 6 and 12 months.

**Table 1:** Outcome measures and delivery time points

Type of data	Outcome measures	Time points				
		1^a^	2^b^	3^c^	4^d^	5^e^
Demographic	Age, gender, ethnic group, age at leaving full-time education, occupation, current work status	Yes				
History	Time since completely free of back pain	Yes				
History	Previous back pain treatments	Yes				
Medications	Current medications	Yes				Yes
History	Satisfaction with health state	Yes				Yes
History	Troublesomeness question	Yes				Yes
Back pain-related disability	Roland Morris disability questionnaire [14]	Yes				Yes
Back pain-related disability	The modified von Korff (MVK) disability score. [15, 16]	Yes				Yes
Back pain severity	MVK pain scale [15]	Yes				Yes
	Modified form of Patient-Generated Index [17, 18]	Yes				Yes
Psychological distress	Depression, Anxiety, and Positive Outlook Scale (DAPOS). [19]	Yes				Yes
Pain self-efficacy	Pain self-efficacy questionnaire [20]	Yes				Yes
Health-related quality of life	SF-12 version 2, reported as physical and mental component scores [21]	Yes				Yes
Health utilities	EuroQol EQ-5D-5 L [22, 23]	Yes		Yes	Yes	Yes
Well-being	Warwick-Edinburgh Mental Well-being Scale (WEMWEBS) [24]	Yes				Yes
Pain distribution	Troublesomeness grid [10]	Yes				
Back pain severity today	11-point pain rating scale [16]		Yes	Yes		
Current work status	If appropriate date of return to work					Yes
Health and social service resource use	Including hospital and community resource, as well as costs to individuals and carers					Yes

^a^1. Baseline - following clinical assessment

^b^2. Intervention only, day of injection 45–60 minutes before and after injection

^c^3. Daily pain score for a period of 35 days starting 7 days before first physiotherapy treatment (via SMS) after which weekly until the endpoint (3 months)

^d^4. EQ-5D-5 L weekly from first physiotherapy treatment session until night before injection appointment when daily for 8 days; then back to weekly until the endpoint (3 months)

^e^5. Follow-up – 3, 6 and 12 months post randomisation

### Procedure and trial interventions

The patient flow through the study is detailed below and illustrated in Fig. 1.

### Participant identification

We will actively identify referrals to secondary care for patients with low back pain and send invitation packs to these patients whilst they are on the waiting list for a clinic appointment. Waiting room posters will allow patients to self-refer to the study. At some sites patients who are attending clinics with low back pain may be approached by their treating clinician or a member of their team, to assess possible participation in the trial. Those patients in which the GP suspects a specific cause for their back pain (tumour, fracture infection, ankylosing spondylitis) will normally have been referred in through a different pathway. Referrals will include, but not necessarily be limited to, pain clinics, neurosurgery, rheumatology and orthopaedic clinics. Potential participants will be sent a brief Information Sheet along with a Screening Questionnaire and Expression of Interest Form by a member of the investigator’s trial team based at the local site (e.g. a research nurse or physiotherapist) to assess preliminary eligibility for enrolment into the trial.

The potential participant will be asked to return the completed Screening Questionnaire and Expression of Interest Form to the study team at the local site in an envelope provided. From review of the questionnaire, if the potential participant appears eligible and interested, a member of the local site trial team will send the full Participant Information Sheet regarding the trial. Specifically the full Information Sheet will inform the patient about the eligibility assessment they are being invited to attend, which will help determine their possible inclusion in the trial, clearly explaining that it is only at this assessment that their eligibility will be confirmed. They will be invited to attend an appointment for their clinical/physical (diagnostic) assessment at a nominated research clinic. Each site will be required to maintain an anonymised trial Screening Log monitoring the number of screening packs sent out, returned and recruited, noting reasons for ineligibility. Only screening data from consenting participants will be returned to the coordination centre at Warwick CTU.

### Diagnostic assessment (visit A)

Those potential participants who are interested and appear eligible based on the Brief Screening Questionnaire and Expression of Interest will be invited to attend an appointment for the initial eligibility physiotherapy assessment. This assessment will be required to confirm eligibility and will be undertaken by a local site research physiotherapist who will not be responsible for undertaking the ‘best usual care’ treatment.

The eligibility assessment appointment will establish patient’s eligibility based on the study’s inclusion/exclusion criteria. The procedures for the diagnostic assessment were informed by the available evidence and a consensus conference of experts [4]. Based on the agreement from the consensus conference, we consider probable facet joint pain to be present when all of the following are true [4]:

1. No radicular symptoms (defined as pain radiating below the knee and no symptoms on a ‘contracted’ neurological testing) [11], and
2. No sacro-iliac joint pain elicited using pain provocation tests, and
3. Increased pain, unilaterally or bilaterally, on lumbar para-spinal palpation, and
4. Increased low back pain on one or more of the following;

–extension (more than flexion)
–rotation
–extension/side flexion^*^
–extension/rotation^*^

^*^Both tests representative of regular compression patterns [12]

A study ‘diagnostic manual’ ensures that all sites adhere to the same procedures when assessing potential participants. Prior to starting the trial, physiotherapists within the investigator site team will be trained in the diagnostic assessment procedures to be used.

At the end of the diagnostic assessment eligible participants will be invited to participate.

### Enrolment procedures

Upon written informed consent, the participant will be scheduled to attend a 1-hour preliminary ‘best usual care’ session delivered by a specially trained physiotherapist. The ‘best usual care’ intervention is outlined below.

### First ‘best usual care’ session (visit B1)

All participants will be scheduled a first 1-hour session with a study-trained physiotherapist. Treatment will follow guidance from NICE [2, 3], but tailored to individual patients. Participants will undergo a thorough initial physical assessment (60 minutes) based on the principles of Maitland manual therapy [13]. The assessment will also include discussion of patient expectations, fear avoidance and perceived self-efficacy.

Once the first trial physiotherapy session is completed, the participant will be randomised to receive facet joint injection with the best usual care package (intervention arm), or the best usual care package only (control arm). The investigator trial team will then complete the randomisation process and inform the participant whether they need to attend for an injection. This will take place within 3 weeks of randomisation.

### ‘Best usual care package’ (visits B2 to B6)

The five remaining sessions will last approximately 30 minutes. All participants (those in the intervention and those in the control arms) will be encouraged to attend all of these sessions. The package is one-to-one sessions with a study physiotherapist who will use the best usual care manual informed by consensus [4]. Sessions will be a bespoke package of physical and behavioural rehabilitation.

All treatment sessions will be complete within 12 weeks of randomisation.

### Facet joint injections (visit C)

For those participants who are randomised to undergo facet joint injection the injection will take place between the first and second best usual care sessions. The treating clinician will at this time make their own assessment of the participant’s suitability for facet joint injection and obtain consent for the procedure following normal practice in each participating Trust. The treating clinician may postpone injection in the presence of short-term illness (e.g. influenza). If significant comorbidity is identified at this time that contra-indicates injection, this will be ‘flagged’ to the patient notes, local investigator, site clinician or general practitioner, whichever is most appropriate. The participant will continue to receive the control intervention.

Up to six facet joints (L3/L4, L4/L5, L5/S1 bilaterally) in each participant will be injected. However, where, on clinical assessment, there is unilateral pain, or involvement of only some levels, the operator may choose to do unilateral injection, or be selective on levels injected. This pragmatic approach reflects current clinical practice within the NHS.

**Fig. 2:**
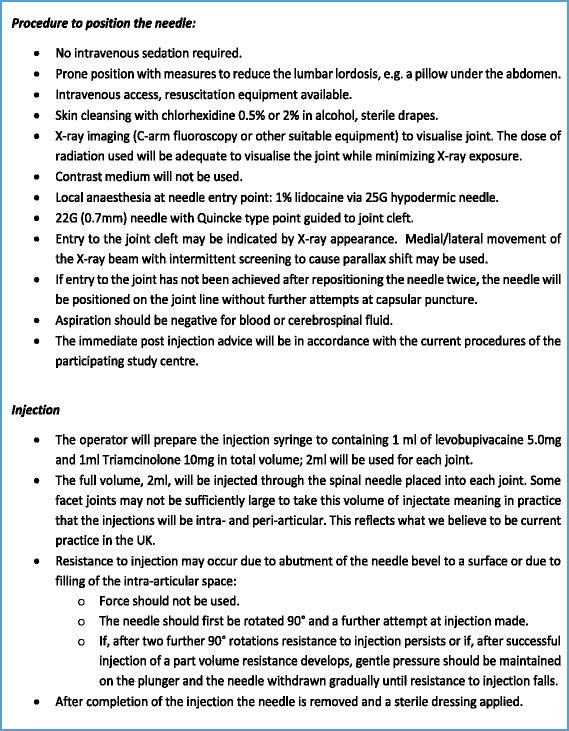
Procedure for intra-articular facet joint injections (for this trial)

Figure 2 outlines the injection procedure that was agreed at consensus [4].

### Economic evaluation

We will use the feasibility data to inform the development of a baseline decision-analytic model, which will allow important elements of resource use, costs and health consequences and gaps in the economic evidence to be identified. This may include an element to account for long-term harms from radiation. Further, we will employ value of information techniques using the data available from the feasibility trial (supplemented, where necessary, using data from secondary sources) to give us an indication of whether it is reasonable to proceed to a main trial. This will supplement our clinical analysis in the decision-making process regarding a main trial. If these data effectively exclude a reasonable possibility that the intervention is cost-effective then we would not wish to proceed to a main trial.

### Process evaluation

Our objectives are to understand the patient and professional participants’ experience of the trial processes, the intervention and intervention sequelae to inform the design of a future full trial.

#### Quantitative data

Data will be collected in relation to the number of patients approached, who agree to participate in the trial, and who are randomised. The number of best usual care sessions run and the number of attendees at each session will be recorded.

#### Participant interviews

A purposive sample of patient participants will be interviewed once they have completed their 3-month follow-up questionnaire. In choosing to do these interviews at this time we are mindful that the interviews themselves might introduce bias into our between-group comparison and that there will be some loss in quality of the qualitative data because of recall bias. Pragmatically, we will do these after we have collected 3 months of follow-up needed for our primary analysis. Interviews will be face to face or by telephone. The choice will be left to the participant to help maximise recruitment. We will recruit for diversity of age, gender and baseline severity of back pain. Interviews will be audio recorded and transcribed. Early interviews will be reviewed and the interview schedule developed based on the data. Recruitment will continue until data saturation. We expect this to be after approximately 15 interviews.

Interviews will cover: past experience of back pain, actions taken to help back pain such as treatments sought, how the participant summarised their experience of back pain as a diary pain score and in response to the EQ-5D-5 L, expectations of the effect of the intervention and whether these were met, any change the participant thinks that has or has not occurred, and experience of trial processes such as recruitment process and use of the diary.

#### Focus groups/interviews with trial team members and clinicians delivering intervention

We will run focus groups (or, if impractical, interviews) with trial team members involved in recruitment and data collection and with clinicians delivering interventions and clinic staff associated with intervention delivery. We will undertake this data collection at each recruiting Trust. Focus groups will be facilitated by an experienced researcher. Where possible they will be observed by a second researcher. Focus groups and interviews will be audio recorded. The topic guide will include: experience of patient recruitment and assessment, intervention delivery, and outcome data collection.

#### Observation of assessment at trial entry and delivery of intervention/control intervention

A researcher independent of the team will sequentially observe up to five of each of: initial assessment, intervention, and control intervention events. The researcher will take field notes and provide almost immediate feedback to the trial team on issues of variation in delivery so these can receive attention. The observations will then inform the development of quality control criteria. The trial team will review and refine draft criteria, which will then be tested on a further five events of each type later in the trial.

### Analysis

We will develop a detailed statistical analysis plan prior to any analyses. Summary statistics will be generated for all variables and data will be presented in tables and charts as appropriate.

The main quantitative outputs from this feasibility trial will, first, be process outcomes assessed for the whole trial population rather than for each treatment group separately and will include:

- Proportion of eligible participants who are randomised and complete follow-up;
- Proportion who obtain immediate (diagnostic) pain relief;
- Recruitment rates; number of referrals per site and proportion of referrals converted to participants. These data are needed to estimate number of sites needed for, and duration of, the main trial;
- Completeness of data from short-term electronic data collection.

Between-group comparisons will be conducted on an intention-to-treat basis, with all participants considered to be in the facet joint injection group if they are randomised to that group even if injections are contra-indicated. We will also perform between-group comparisons of the following outcomes on an intention-to-treat basis:

- Average pain, as measured using text messaging (or paper back-up), over 3 months;

AND

- Back pain-related disability, as measured using Roland Morris disability questionnaire (RMDQ), reported at 3-month follow-up.

If it is not feasible to recruit sufficient participants to make these between-group analyses meaningful they will omitted.

We will adjust our models for baseline stratification factors and other baseline covariates.

#### Analysis of qualitative data

The objectives of the process evaluation will provide the framework for analysis of the interview transcripts, observation field notes and focus group transcripts. We will undertake a thematic analysis, interrogating and coding the data for each of the objectives. Data relevant to each theme will be compared within samples (patients/observed events/focus groups) paying particular attention to variation in the data from participants with different characteristics (e.g. age) or from different contexts. Data relevant to each theme will also be compared across samples e.g. contrasting patient/clinician data. The analysis will be written up as a report to inform the design of a subsequent main trial.

Data will be synthesised and anonymous quotations will be used as exemplars of themes.

### Regulatory authorities/ethical approval

The Facet Injection Study comes under the definition of a Clinical Trial of an Investigational Medicinal Product (CTIMP) under the EU Clinical Trials Directive 2001/20/EC and will therefore require submission to the Medicines and Healthcare Products Regulatory Agency (MHRA). In accordance with ICH E6-GCP ethical approval was obtained on 20 August 2014 (Committee Yorkshire and the Humber – Sheffield, REGO 2013-592). MHRA approval was obtained on 18 November 2014 (ref. 13268/0001/001-0001, Eudract number: 2014-000682-50).

### Investigational medicinal product (IMP)

A study ‘injection manual’ will be prepared to ensure that all sites adhere to the same procedures when delivering the facet joint injection.

The IMP 1 ml of levobupivacaine 5.0 mg/ml and 1 ml of triamcinolone 10 mg/ml prepared within the same syringe will be used for each facet joint injection procedure. Preparation of the injection will be undertaken by the operator immediately prior to the injection. A total volume of 2 ml will be injected through the spinal needle placed into each joint (see Fig. 2). This approach of mixing such drugs immediately prior to facet joint injection is standard practice for this procedure within the NHS.

### Drug storage and dispensing and drug accountability

The IMP used will be the usual drug provided for use by the participating Trust’s pharmacy departments. Appropriate procedures will be established and monitored to ensure that the drugs are correctly stored, dispensed and accounted for. The study team will adhere to appropriate standard operating procedures and legislation for trials of IMPs.

### Adverse event management

Adverse events will be managed in line with Warwick CTU’s standard operating procedures

### Adverse events (AEs)

We expect that participants will experience some uncomfortable effects from participation in the ‘best usual care’ treatment – for example muscle or joint soreness in response to exercise, feeling unwell or anxious. These and similar effects are entirely to be anticipated, and provided they are short-lived or dealt with through clinical management should not be reported as adverse events.

The following are expected adverse events and will be recorded in the case report form (CRF):

- Pain, bleeding, discomfort and minor bruising at the injection site (transient)
- Numbness in the buttocks and legs from local anaesthetic (transient)
- Infection of injection site (uncommon)
- Inadvertent intravenous injection (uncommon)
- Musculoskeletal injuries requiring medical attention including serious sprains, joint dislocation, falls or other injuries occurring as a direct consequence of the intervention (i.e. whilst participating in the ‘best usual care’ physiotherapy treatment intervention in real-time) should also be recorded.

All serious adverse events (SAEs) and suspected unexpected serious adverse events (SUSARs) that occur between trial entry and up to the end of the final ‘best usual care’ physiotherapy treatment session will be entered onto the SAE reporting form within the participant’s CRF. Reporting of SAEs and SUSARs will follow Warwick Clinical Trials Unit’s standard operating procedures and legislation.

### Data storage and management

Data will be managed in line with WCTU’s standard operating procedures

### Trial management

Day-to-day management of the trial is carried out by a trial coordinator at WCTU, they are managed by a senior project manager and the chief investigator. There are regular meetings of the trial management group. As the trial has a short recruitment time it was agreed with the funder that a Data Monitoring and Ethics Committee (DMEC) was not necessary. An independent Trial Steering Committee (TSC) was appointed by the funder.

### CONSORT

The trial will be reported in line with the Consolidated Standards of Reporting Trials (CONSORT) statement (Lancet 2001, 357: 1191–4). In accordance with reporting guidlines a SPIRIT checklist is included as an Additional file 2.

## Discussion

This feasibility trial will be one of the larger trials exploring the use of therapeutic intra-articular facet joint injections yet undertaken. Trials like this are not without their challenges not least that this is a controversial area of research. It is important, whatever the outcome, that this trial provides robust findings, which can inform the future of work in this area. With this in mind we have developed a protocol based on the current best evidence and through consensus with experts.

### Trial status

The study is currently in the early stages of recruitment.

## Additional files

Additional file 1:
**Web appendix facet_joint_sample_size.** (PDF 34 kb) Additional file 2:
**SPIRIT_checklist.** (DOCX 48 kb)

## Abbreviations

AE: Adverse events
CONSORT: Consolidated Standards of Reporting Trials
COPD: Chronic obstructive pulmonary disease
CRF: Case report form
DMEC: Data Monitoring and Ethics Committee
GP: General practitioner
IMP: Investigational medicinal product
MHRA: Medicines and Healthcare Products Regulatory Agency
NHS: National Health Service
NICE: National Institute for Health and Care Excellence
RMDQ: Roland Morris disability questionnaire
SAEs: Serious adverse events
SUSARs: Suspected unexpected serious adverse events
TSC: Trial Steering Committee
WCTU: Warwick Clinical Trials Unit

## References

[1] Maniadakis N, Gray A (2000). The economic burden of back pain in the UK. Pain..

[2] National Institute for Health and Care Excellence (2009). Low back pain: early management of persistent non-specific low back pain.

[3] Savigny P, Watson P, Underwood M (2009). Early management of persistent non-specific low back pain: summary of NICE guidance. BMJ..

[4] Mars T, Ellard DR, Antrobus JH, Cairns M, Underwood M, Haywood K (2015). Intraarticular facet injections for low back pain: design considerations, consensus methodology to develop the protocol for a randomized controlled trial. Pain Physician..

[5] Falco FJ, Manchikanti L, Datta S, Sehgal N, Geffert S, Onyewu O (2012). An update of the systematic assessment of the diagnostic accuracy of lumbar facet joint nerve blocks. Pain Physician..

[6] Manchikanti L, Abdi S, Atluri S, Benyamin RM, Boswell MV, Buenaventura RM (2013). An update of comprehensive evidence-based guidelines for interventional techniques in chronic spinal pain. Part II: guidance and recommendations. Pain Physician.

[7] Manchikanti L, Singh V, Falco FJ, Cash KA, Fellows B (2010). Comparative outcomes of a 2-year follow-up of cervical medial branch blocks in management of chronic neck pain: a randomized, double-blind controlled trial. Pain Physician..

[8] Jack K, McLean SM, Moffett JK, Gardiner E (2010). Barriers to treatment adherence in physiotherapy outpatient clinics: a systematic review. Manual Ther..

[9] NETSCC. HTA - 11/31/01: Facet Feasibility (FF) Southampton: National Institute for Health Research; 2015[cited 2015 8th Sept]. Available from: http://www.nets.nihr.ac.uk/projects/hta/113101.

[10] Parsons S, Carnes D, Pincus T, Foster N, Breen A, Vogel S (2006). Measuring troublesomeness of chronic pain by location. BMC Musculoskelet Disord..

[11] McCarthy C, McCarthy C (2010). Neurological assessment. Combined movement theory rotational mobilization of the vertebral column.

[12] Edwards B (1999). Manual of combined movements: their use in the examination and treatment of musculoskeletal vertebral column disorders.

[13] Maitland G, Hengeveld E, Banks K, English L (2005). Maitland's vertebral manipulation.

[14] Roland M, Morris R (1983). A study of the natural history of back pain. Part I: development of a reliable and sensitive measure of disability in low-back pain. Spine.

[15] Underwood MR, Barnett AG, Vickers MR (1999). Evaluation of two time-specific back pain outcome measures. Spine..

[16] Von Korff M, Ormel J, Keefe FJ, Dworkin SF (1992). Grading the severity of chronic pain. Pain..

[17] Klokkerud M, Grotle M, Lochting I, Kjeken I, Hagen KB, Garratt AM (2013). Psychometric properties of the Norwegian version of the patient generated index in patients with rheumatic diseases participating in rehabilitation or self-management programmes. Rheumatology (Oxford).

[18] Løchting I, Grotle M, Storheim K, Werner E, Garratt A (2014). Individualized quality of life in patients with low back pain: reliability and validity of the Patient Generated Index (PGI). J Rehabil..

[19] Pincus T, Rusu A, Santos R (2008). Responsiveness and construct validity of the depression, anxiety, and positive outlook scale (DAPOS). Clin J Pain..

[20] Nicholas MK (2007). The pain self-efficacy questionnaire: taking pain into account. Eur J Pain..

[21] Ware J, Kosinski M, Keller SD (1996). A 12-Item Short-Form Health Survey: construction of scales and preliminary tests of reliability and validity. Med Care..

[22] The EuroQol Group (1990). EuroQol--a new facility for the measurement of health-related quality of life. Health Policy.

[23] Brazier J, Roberts J, Deverill M (2002). The estimation of a preference-based measure of health from the SF-36. J Health Econ..

[24] Tennant R, Hiller L, Fishwick R, Platt S, Joseph S, Weich S (2007). The Warwick-Edinburgh Mental Well-being Scale (WEMWBS): development and UK validation. Health Qual Life Outcomes..

