# Supplementary material for: Facet-joint injections for people with persistent non-specific low back pain (FIS): study protocol for a randomised controlled feasibility trial
Source: Trials. 2015 Dec 24;16:588. doi: 10.1186/s13063-015-1117-z (PMC4690389; doi:10.1186/s13063-015-1117-z)
Supplement: Additional file 1: — Web appendix facet_joint_sample_size. (PDF 34 kb) [file 13063_2015_1117_MOESM1_ESM.pdf]

Facet feasibility study sample size calculations

At the end of the feasibility trial, we will obtain an estimate, $\hat{\theta}$ of the unknown treatment difference along with a 95% confidence interval given by

$$\left(\hat{\theta} - 1.96\sqrt{\frac{2\sigma^2}{n/2}}, \hat{\theta} + 1.96\sqrt{\frac{2\sigma^2}{n/2}}\right).$$

We will consider proceeding to the main study if the upper limit exceeds some prescribed target δ.

For a true treatment difference of θ, we have , $\hat{\theta} \sim N(\theta, 2\sigma^2/(n/2))$, so that

$$pr\left(\hat{\theta} + 1.96\sqrt{\frac{2\sigma^2}{n/2}} \geq \delta; \theta\right) =$$

$$pr\left(\hat{\theta} \geq \delta - 1.96\sqrt{\frac{2\sigma^2}{n/2}}; \theta\right) =$$

$$1 - \Phi\left(\frac{\delta - 1.96\sqrt{\frac{2\sigma^2}{n/2}} - \theta}{\sqrt{\frac{2\sigma^2}{n/2}}}\right).$$

Calculation of this expression gives the probability of recommending progression to the main study with total sample size n when the true treatment effect is θ.

If the true treatment effect is equal to the specified target value, that is $\theta = \delta$, this probability is $1 - \Phi(1.96) = 0.975$, so that the probability is 0.975 irrespective of the sample size or choice of δ (this follows from the definition of a 95% confidence interval). For $\theta = 0$, we get the probability of erroneously proceeding to the main study when the true effect is zero. Values for a range of values of n and δ are given in the Table 1 below.

These probabilities can be interpreted in a number of ways. The test can be viewed as a test of the null hypothesis $\theta = \delta$ conducted at one-sided 2.5% level. The probabilities in Table 1 then correspond to the power of this test to detect the alternative $\theta = 0$.

Alternatively, considering a conventional test of the null hypothesis that $\theta = 0$, this approach fixes the power to detect a difference of size δ to be 97.5% (and does so irrespective of the unknown variance). The type I error rates is then determined by the sample size (and σ^2), and is given by the values in Table 1.

The approach of using a high power and a high type I error rate in pilot studies has been advocated by a number of authors (see, for example, Schoenfeld, 1980, Stallard, 2012)

Table 1: Probability of deciding to proceed to main study when true treatment effect is 0 for range of sample size and δ values

δ	$n = 80$	$n = 90$	$n = 100$	$n = 110$	$n = 120$	$n = 130$	$n = 140$
0.2	0.857	0.844	0.831	0.819	0.806	0.794	0.781
0.25	0.800	0.781	0.761	0.742	0.723	0.704	0.685
0.3	0.732	0.704	0.677	0.651	0.624	0.599	0.573
0.35	0.653	0.618	0.583	0.550	0.517	0.486	0.456
0.4	0.568	0.525	0.484	0.445	0.409	0.374	0.342

It is proposed to take n to be approximately 130 (150 less about 20% dropouts). For a δ of 0.35, this gives a probability of proceeding to the main study when the true treatment effect is zero of approximately 50%. Equivalently, this decision will be taken provided the treatment effect estimate at the end of this study is at least positive.

References

Schoenfeld, D. Statistical considerations for pilot studies. *International Journal of Radiation Oncology, Biology and Physics* 1980; **6**: 371-374.

Stallard, N. Optimal sample sizes for phase II clinical trials and pilot studies. *Statistics in Medicine* 2012; **31**: 1031-1042.
